# Reframing small cell lung cancer: therapeutic lessons from hematologic malignancies

**DOI:** 10.3389/fonc.2026.1821271

**Published:** 2026-04-30

**Authors:** Kanak Parmar, Ira Surolia, Anish Thomas

**Affiliations:** 1 National Heart, Lung, and Blood Institute (NHLBI), National Institutes of Health (NIH), Bethesda, MD, United States; 2 Hartford Healthcare, Hartford, MD, United States; 3 Developmental Therapeutics Branch, National Cancer Institute (NCI), National Institutes of Health (NIH), Bethesda, MD, United States

**Keywords:** antibody drug conjugates, antigen-directed therapy, biomarkers, bispecific T cell engagers, circulating tumor DNA, lung cancer, DNA damage response, epigenetic priming

## Abstract

Small cell lung cancer (SCLC) remains one of the most aggressive malignancies, characterized by rapid proliferation, early metastatic dissemination, and poor long-term survival despite initial sensitivity to therapy. SCLC shares several biologic and therapeutic principles with hematologic malignancies, including lineage state dependence, adaptive resistance through non-genetic plasticity, and emerging susceptibility to antigen-directed immune therapies. Reframing SCLC through this lens provides a conceptual framework for understanding treatment failure and identifying new therapeutic strategies. A defining feature of SCLC is its dynamic transition between neuroendocrine (NE) and non-neuroendocrine (non-NE) states, driven by epigenetic and transcriptional reprogramming rather than new genetic alterations. These state transitions regulate antigen expression, immune visibility, and therapeutic vulnerability, enabling tumors to evade both cytotoxic and immune-based treatments. This plasticity parallels lineage switching and antigen escape observed in hematologic malignancies treated with targeted and immune therapies. Recent advances in antigen-directed therapy, particularly bispecific T cell engagers and antibody-drug conjugates targeting lineage-associated proteins such as DLL3, SEZ6, and TROP2, have demonstrated promising clinical activity. However, therapeutic efficacy is limited by antigen heterogeneity, evolving tumor states, and microenvironmental barriers including immune exclusion and T cell dysfunction. Epigenetic therapies targeting regulators such as EZH2 and LSD1 offer a strategy to reprogram tumor state, enhance antigen presentation, and sensitize tumors to immunotherapy. Beyond lineage biology, SCLC exhibits dependence on replication stress and DNA damage response pathways, though targeting these vulnerabilities alone has yielded modest clinical benefit. Emerging evidence highlights the role of metabolic and stress-response adaptations, including lactate-mediated immune suppression and integrated stress signaling, in sustaining tumor fitness and resistance. Circulating tumor DNA and epigenomic profiling provide noninvasive approaches to monitor tumor evolution, lineage state, and treatment response over time, offering potential for biomarker-guided therapeutic adaptation. Overall, durable clinical benefit in SCLC will likely require temporally sequenced, biomarker driven combination strategies that anticipate and constrain tumor plasticity. Integrating lineage-directed targeting, epigenetic modulation, immune engagement, and metabolic intervention may enable more effective and sustained disease control in this highly adaptive cancer.

## 1 Introduction

Small cell lung cancer (SCLC) accounts for approximately 10-15% of all lung cancers and is strongly linked to tobacco exposure (1, 2). In the United States, SCLC incidence has declined steadily over the past two decades, paralleling reductions in smoking prevalence. Nevertheless, it continues to represent a substantial clinical burden, with an estimated 250,000 new cases and at least 200,000 deaths globally each year (3). The disease typically presents in older adults, with a median age at diagnosis in the late 60s, and is characterized by aggressive clinical behavior, rapid growth, and early metastatic dissemination.

Historically considered a purely smoking-induced cancer, SCLC is increasingly understood as a disorder of defective genome maintenance. Beyond mutagenic tobacco exposure, a subset of patients harbor pathogenic germline variants, most commonly affecting DNA damage response pathways (4). These include alterations in homologous recombination and checkpoint signaling genes such as *CHEK1*, *BRCA2*, and *MUTYH* (5). Together with pervasive somatic alterations in *TP53* and *RB1*, these findings support a model in which replication stress and impaired DNA repair are foundational features of SCLC biology.

Although the addition of immune checkpoint inhibitors (ICI) to the carboplatin-etoposide backbone has improved survival, the magnitude of benefit remains limited: the 5-year survival for SCLC is approximately 9% (6). Nonetheless, therapeutic progress in SCLC is accelerating, driven in part by its designation as a recalcitrant cancer (7). A major barrier to durable therapy is the reversible transcriptional states of SCLC. Tumors dynamically transition among neuroendocrine (NE) and non-neuroendocrine (non-NE) programs, altering antigen expression, immune visibility, and drug sensitivity in the absence of new genetic alterations.

These properties, state dependent phenotypes, adaptive antigen modulation, and relapse through cellular reprogramming, parallel organizing principles long recognized in hematologic malignancies. SCLC also shares several clinical features with aggressive hematologic malignancies, including rapid proliferative kinetics, bone marrow involvement, high initial responsiveness to cytotoxic therapy, and rapid emergence of treatment resistance (8, 9). In this review, we therefore consider SCLC through a hematopoietic lineage-based framework, examining how lineage plasticity, antigen heterogeneity, immune context, and stress response dependencies collectively shape both vulnerability and resistance. We discuss therapeutic strategies designed to constrain these adaptive programs. Finally, we draw parallels from mechanisms of resistance to T-cell engaging therapies, long used in hematologic malignancies to inform the rational use and sequencing of these agents in SCLC.

## 2 Shared biological features of hematologic malignancies and SCLC

Accumulating evidence positions SCLC within a broader pan cancer convergence toward a lineage-restricted small cell NE phenotype that shares core therapeutic susceptibilities with hematologic malignancies (10). A key shared feature is reliance on anti-apoptotic BCL-2 family proteins to prevent cell death, together with dependence on DNA damage and replication-stress pathways to support rapid proliferation despite genomic instability (10, 11). However, clinical trials targeting these dependencies have yielded inconsistent and generally short-lived responses, in part because anti-apoptotic dependence is heterogeneous and plastic (e.g., shifting between BCL-2, BCL-XL, and MCL-1), and single-agent inhibition is often bypassed by state-dependent redundancy and adaptive rewiring (12–14). Another potential parallel with hematologic malignancies is RUNX1T1-associated epigenetic regulation (15).

Beyond these cell intrinsic dependencies, emerging transcriptional subtypes reveal functional parallels with hematologic malignancies. Non-NE or inflamed SCLC states are enriched for immune-related programs, including antigen presentation and interferon signaling (16). While immune engagement is not unique to hematologic cancers, malignant cells in leukemias and lymphomas arise and persist within immune-rich niches and are continuously shaped by immune surveillance. In this respect, inflamed SCLC states resemble aspects of hematologic malignancies, where disease behavior and treatment response are tightly coupled to immune context (17–19).

Another, clinically important parallel is that cell state often determines therapeutic vulnerability. In hematologic malignancies, long-term control is achieved by therapies that shift the disease into a more vulnerable state, as seen in TP53-mutated acute myeloid leukemia (AML)/myelodysplastic syndrome (MDS), where the depth of initial cytoreduction does not reliably predict durability of response (20). Conventional DNA-damaging therapies frequently fail because leukemic cells tolerate genotoxic stress without activating p53 dependent apoptosis (21). In contrast, hypomethylating agents such as decitabine exert clinical activity primarily through DNMT1 depletion and epigenetic remodeling rather than acute cytotoxicity, effectively reprogramming tumor state (22).

SCLC may present an analogous opportunity for state directed therapy. Epigenetic regulators such as EZH2 are frequently upregulated and functionally important in SCLC (23, 24). Across tumor types, polycomb-mediated repression constrains immunogenic gene programs, providing a mechanistic rationale for epigenetic priming in immune-evasive SCLC (23, 25). Supporting this concept, a clinical study of the EZH1/2 inhibitor valemetostat demonstrated on-treatment increases in MHC-I expression and evidence of subtype switching in recurrent SCLC, consistent with epigenetic modulation of tumor state (26).

Collectively, these observations suggest that durable benefit in SCLC will likely depend on temporally sequenced, state-guided combination strategies that exploit therapy induced vulnerabilities before adaptive plasticity restores tumor equilibrium.

## 3 Lineage dependency as a therapeutic vulnerability

**Figure 1:**
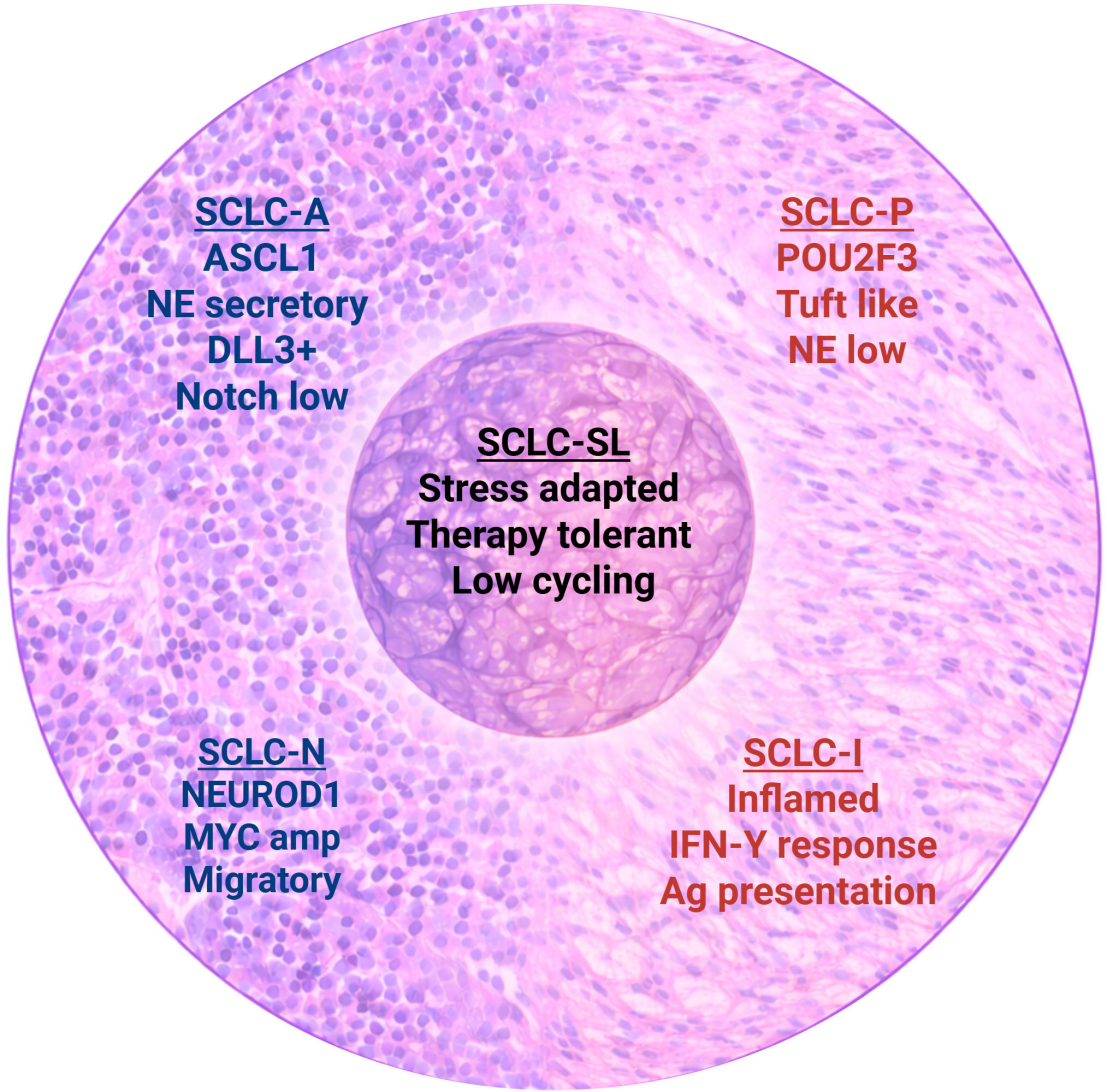
Transcriptional states and plasticity in SCLC. Schematic representation of major SCLC lineage states. NE-high states (SCLC-A and SCLC-N) are characterized by ASCL1 or NEUROD1 lineage programs and associated features including DLL3 expression. Non-NE states (SCLC-P and SCLC-I) display tuft cell– like or inflammatory gene signatures and interferon/antigen presentation pathways. The central compartment SCLC-stem like (SCLC-SL) state represents a stress adapted, therapy tolerant, low cycling plastic state, that may enable dynamic transitions between NE and non-NE programs under selective pressure.

SCLC is organized into lineage defined NE and non-NE transcriptional states that impose distinct surface antigen repertoires, signaling dependencies, and therapeutic susceptibilities (27–29) (**Figure 1**).

Expression of surface antigens such as delta-like ligand 3 (DLL3) and seizure-related homolog protein 6 (SEZ6) is tightly coupled to activation of the NE gene regulatory network, driven primarily by ASCL1 and, in a subset of tumors, NEUROD1 (29, 30). Maintenance of the NE state depends on coordinated epigenetic regulation, including LSD1 (KDM1A) mediated chromatin control, EZH2-dependent PRC2 repression of alternative lineage programs, and SWI/SNF-regulated enhancer accessibility, which together reinforce ASCL1-centered transcriptional output (31, 32). These chromatin programs operate within a developmental signaling context defined by suppressed canonical Notch activity, further reinforced by DLL3-mediated Notch inhibition, thereby preserving expression of NE associated surface antigens (33, 34).

**Figure 2:**
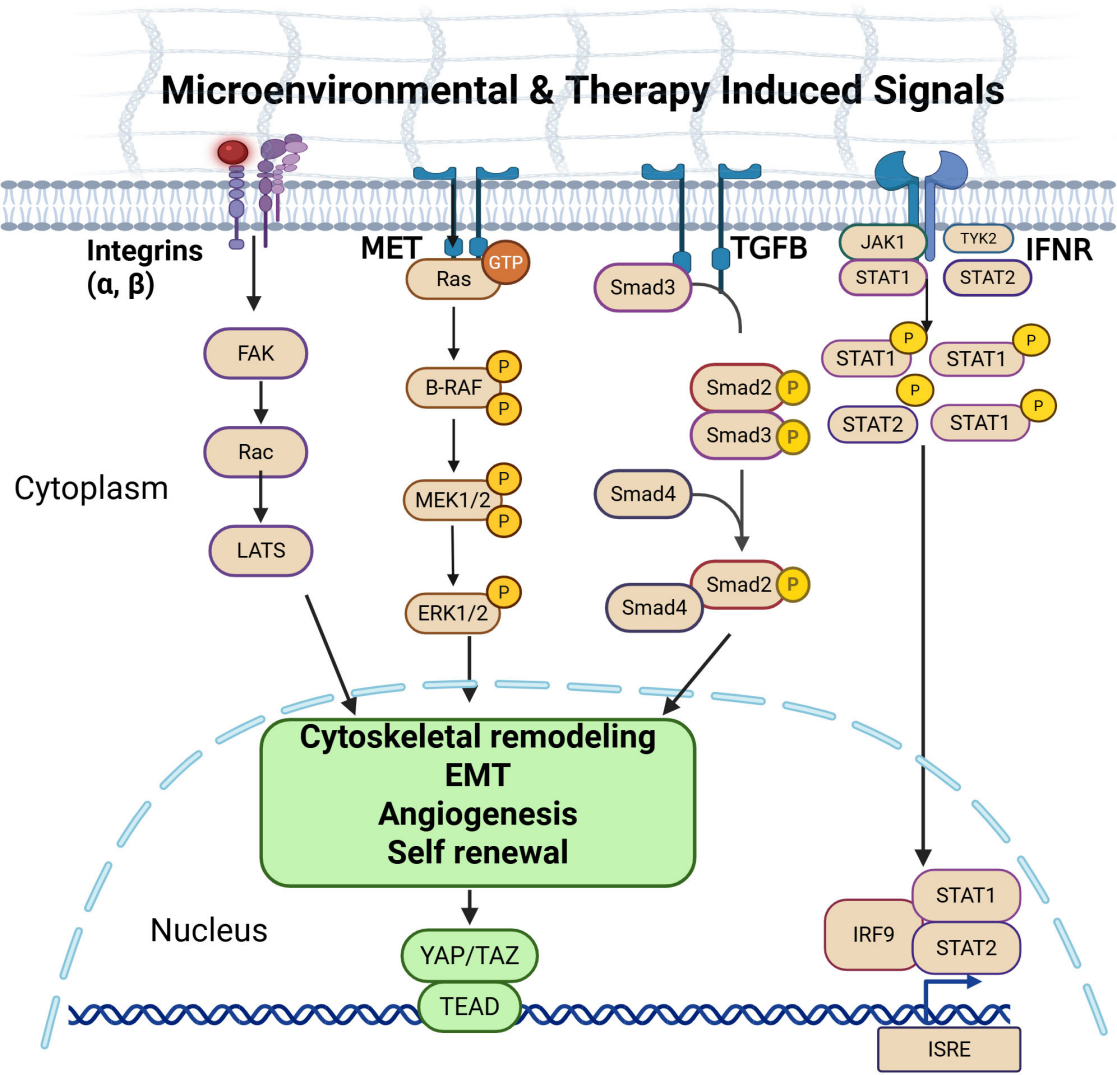
Therapy induced signaling pathways driving lineage plasticity in SCLC. Therapeutic pressure induces adaptive activation of receptor tyrosine kinases (RTKs) and cytokine pathways, including FAK, AXL, MET, TGF-β, and interferon/JAK–STAT signaling, which converge on cytoskeletal remodeling, EMT-associated programs, and transcriptional stress responses. These signaling inputs promote therapy-induced enhancer rewiring, enabling redistribution of chromatin accessibility and facilitating transition away from stable NE identity. Activation of YAP/TAZ/TEAD transcriptional programs functions as a key executor of this plastic state, driving angiogenesis and self-renewal.

In contrast, loss of the NE program and emergence of non-NE states are driven by coordinated activation of lineage reprogramming networks. MYC family activity promotes departure from ASCL1-high differentiation by reshaping enhancer landscapes and redistributing chromatin accessibility between NE and alternative lineage genes (33, 35). Activation of the Hippo pathway, via YAP TEAD transcriptional output, serves as a central effector of the non-NE phenotype, promoting EMT-like features, cellular motility, and pro-inflammatory cytokine production that reshape the tumor microenvironment (36). YAP upregulation may also contribute to acquired therapeutic resistance. Preclinical studies suggest that YAP activation promotes transition from NE to non-NE states, a shift associated with chemoresistance and increased cellular plasticity (36, 37). These observations support discussion of YAP associated lineage reprogramming as one mechanism by which resistant SCLC samples evolve under treatment pressure. Concomitantly, enrichment of interferon/JAK–STAT signaling and TGF-B associated programs is frequently observed alongside reduced NE lineage activity, contributing to stress responsive transcriptional states, extracellular matrix remodeling, and immune modulation (38–40). (**Figure 2**).

Rather than representing fixed or binary subtype transitions, these lineage programs generate dynamic intermediary states characterized by epigenetic plasticity and diminished lineage commitment. This regulatory architecture is exemplified by the SCLC-SL (stem-like) subtype, which captures a broadly dedifferentiated, immune-excluded state associated with inferior survival across clinical cohorts (41). Under therapeutic pressure, this plastic compartment can expand, giving rise to stress-adapted tumor cells capable of toggling between NE and non-NE regulatory programs. These reprogrammed SCLC-SL cells exhibit enhanced metabolic flexibility, MYC-driven transcriptional output, oxidative stress tolerance, and immune evasiveness, collectively enabling persistence despite cytotoxic and immune-based therapies (41–43). An updated list of ongoing trials in small cell lung cancer is provided in **Table 1**.

**Table 1:** Ongoing clinical trials in SCLC across lineage-directed, immune-redirecting, and state-adaptive therapeutic strategies.

Table 1: Ongoing clinical trials in SCLC.				
Treatment Class	Agent	Target	Stage	Trial
A. Lineage-Restricted Antigen Targeting (Payload-Dependent)				
ADC	ZL-1310	DLL3	Phase I	NCT06179069
ADC	IBI-3009	DLL3	Phase I	NCT06613009
ADC	ABBV-011	SEZ6	Phase I	NCT03639194
ADC	ABBV-706	SEZ6	Phase I	NCT05599984
B. Lineage-Constrained Immune Vulnerabilities				
TCE	ZG006	DLL3 × CD3	Phase I/II	NCT05978284
TCE	BI764532	DLL3 × CD3	Phase I/II	NCT05879978
TCE	MK-6070 (HPN328)	DLL3 × CD3	Phase I/II	NCT06780137
TCE	QLS31904	DLL3 × CD3	Phase I	NCT05461287
TCE	RG-6524 (RO7616789)	DLL3 × CD3 × CD137	Phase I	NCT05619744
TCE	Tarlatamab	DLL3 × CD3	Phase I–III	DeLLphi
II. Cellular Therapies in Highly Selected Lineage-Positive Disease				
CAR-T	AMG 119	DLL3	Phase I	NCT03392064
TriTAC	HPN328	DLL3	Phase I	NCT04471727
CAR-T	LB2102	DLL3	Phase I	NCT05680922
CAR-NK	SNC115	DLL3	Phase I	NCT06384482
III. Payload-Driven Cytotoxicity Across Lineage States				
A. Immune-Evasive				
ADC	Ifinatamab-DXd	B7-H3	Phase III	NCT06612151
ADC	YL201	B7-H3	Phase III	NCT06612151
ADC	MGC018	B7-H3	Phase II	NCT06227546
B. EMT-Like/Therapy-Adapted Non-NE States				
ADC	Sac-TMT	TROP2	Phase I/II	NCT04152499
C. Epigenetically Inducible or Heterogeneous Antigens				
ADC	M3554	GD2	Phase I	NCT06641908
Radioligand	GD2-SADA	GD2	Phase I	NCT05130255
IV. Radiopharmaceutical Approaches				
Radioligand	ABD147	Actinium-225	Phase I	NCT06736418
V. Replication Stress and DNA Damage Response Dependencies				
PARP + alkylator	Talazoparib + Temozolomide		Phase I/II	NCT03672773
PARP + XPO1	Olaparib + Selinexor		Phase I/II	NCT05975944
DNA-alkylator	Palifosfamide-tris		Phase III	NCT01555710
Top-I	JK-1201I		Phase III	NCT06581380
VII. Microenvironmental and Stromal Modulation				
Bispecific	BNT327	PD-L1 × VEGFA	Phase III	NCT06712355
TKI	Anlotinib	VEGF	Phase IV	NCT06931145
Bispecific	PT217	DLL3 × CD47	Phase II	NCT06478043
Peptide	LY2510924	CXCR4	Phase II	NCT01439568
VIII. Immune Checkpoint and Glycan-Directed Therapies				
ICI	Vibostolimab	TIGIT	Phase III	NCT05224141
mAb	Adebrelimab	PD-L1	Phase II	NCT06236997
mAb	BMS-986012	fucosyl-GM1	Phase II	NCT04702880
mAb	BMS-986489	fucosyl-GM1	Phase II	NCT06773910
Bispecific	QL1706	PD-1 × CTLA-4	Phase II	NCT05309629
mAb	Tifcemalimab	BTLA	Phase III	NCT06077240
mAb	Monalizumab	NKG2A	Phase II	NCT05121221
IX. Epigenetic and Developmental Pathway Modulation				
EZH2 inhibitor	Valemetostat	EZH2	Phase I	NCT06807632
HMA	Guadecitabine	DNMT	Phase II	NCT03913455
Hedgehog	GDC-0449	SMO	Phase II	NCT00887159
Hedgehog	BMS-833923	SMO	Phase IB	(NCT00927875
Multi-TKI	AL3810	VEGFR/FGFR/PDGFR	Phase II/III	NCT04254471

Agents and regimens are organized by therapeutic class and biological rationale (e.g., lineage-restricted antigen targeting, immune redirection, cellular therapies, payload-driven cytotoxicity across lineage states, radiopharmaceutical approaches, DNA damage response/replication-stress targeting, microenvironmental modulation, immune checkpoint/glycan-directed therapies, and epigenetic/developmental pathway modulation). For each entry, the modality, lead agent, target, and current clinical development stage are listed with the corresponding ClinicalTrials.gov identifier (NCT). Trial status, phase, and design elements reflect information available on ClinicalTrials.gov at the time of table compilation and may change with ongoing updates.

### 3.1 NE lineage markers

#### 3.1.1 DLL3

DLL3 is selectively overexpressed on SCLC tumor cells and largely absent from normal adult tissues, making it an attractive lineage-restricted therapeutic target (44). Early efforts focused on antibody drug conjugates (ADC), notably rovalpitumab tesirine (Rova-T) (45). However, phase II/III studies failed to demonstrate survival benefit and revealed substantial toxicity, including pleural effusions, photosensitivity, anorexia, and dyspnea (46–48). These adverse events (AEs) were attributed to premature release of the pyrrolobenzodiazepine payload and off tumor effects (48). A next-generation DLL3 ADC, zocilurtatug pelitecan (ZL-1310), is currently being evaluated in a randomized phase III trial in relapsed SCLC (NCT07218146) (49, 50).

Unlike ADCs, which rely on intracellular payload delivery, T cell engagers (TCE) redirect endogenous T cell cytotoxicity and can mediate tumor regression independent of target internalization efficiency (6). Tarlatamab (AMG 757) is a first-generation DLL3 directed T cell engager which induces localized T-cell recruitment and cytokine release. In a phase III randomized trial, tarlatamab improved overall survival compared with chemotherapy (median OS 13.6 vs 8.3 months; HR 0.6), with modest progression-free survival improvement (4.2 vs 3.7 months) and higher objective response rate (35% vs 20%) (51). Responses were both rapid and durable, leading to regulatory approval and establishing DLL3-directed T cell redirection as a validated therapeutic strategy in relapsed SCLC.

Tarlatamab is associated with a toxicity profile characteristic of T cell redirecting therapies, cytokine release syndrome (CRS) and immune effector cell associated neurotoxicity syndrome (ICANS). In the DeLLphi-304 study, CRS occurred in 56% of patients, with grade ≥3 CRS observed in 1% patients. Neurologic toxicity was also reported in 56% of patients in the pooled safety population, with ICANS in 6%, including one grade 5 event (52). By contrast, an early real world retrospective series that included patients with untreated brain metastases demonstrated substantially higher first-cycle immune toxicity, with CRS in 72.7% and ICANS in 40.9%, particularly among those with active intracranial disease (53).

Given these risks, U.S. prescribing information recommends step-up dosing of tarlatamab with monitoring in an appropriate healthcare setting for 22–24 hours after the cycle 1 day 1 and day 8 infusions (54). Management strategies are based on established consensus frameworks for cellular therapies and bispecific antibodies, with CRS and ICANS graded and treated according to American Society for Transplantation and Cellular Therapy criteria (55). Low grade CRS is typically managed with supportive care, whereas higher-grade events require IL-6 receptor blockade (e.g., tocilizumab) with or without corticosteroids. Corticosteroids are the primary therapy for ICANS. Available clinical experience indicates that guideline directed use of steroids or IL-6 blockade does not compromise antitumor efficacy (56).

Additional DLL3 targeted TCE are under evaluation. Obrixtamig, a DLL3/CD3 IgG-like bispecific, has demonstrated encouraging early activity in combination with topotecan after platinum therapy, with an unconfirmed response rate of 76% and median progression-free survival of approximately 8 months (NCT05990738) (57). Gocatamig has likewise shown promising activity in early studies (objective response rate ~46%) and is being explored in combination with ifinatamab deruxtecan, a B7-H3 directed ADC, as well as in regimens incorporating checkpoint blockade (NCT07227597; NCT06780137).

Ongoing efforts aim to optimize safety while enabling broader outpatient delivery, including refinement of monitoring practices, improved patient selection, and identification of early biomarkers predicting toxicity risk (58, 59). As DLL3-directed bispecific therapies continue to evolve, standardization of mitigation strategies will be essential to minimize inpatient resource utilization without compromising safety.

Beyond ADCs and T-cell engagers, other DLL3-directed therapeutic platforms are also under investigation. Adoptive cellular therapies targeting DLL3 have shown potent activity in preclinical models but face translational challenges in SCLC, including limited T cell trafficking, an immunosuppressive tumor microenvironment, and potential on-target off-tumor toxicity (60, 61). Strategies to overcome these barriers include cytokine-armored CAR-T cells, such as IL-18 secreting constructs designed to enhance persistence and function (62). In parallel, DLL3-directed radioimmunotherapy has demonstrated early signals of antitumor activity with limited toxicity, further expanding the therapeutic landscape of lineage directed targeting in SCLC (63).

#### 3.1.2 SEZ6

SEZ6 has emerged as a novel cell surface antigen in SCLC, preferentially expressed in NE lineage tumors and closely linked to ASCL1 driven transcriptional programs (29). ABBV-011, a first generation SEZ6 targeting ADC conjugated to a calicheamicin payload, was evaluated in a first-in-human phase I trial that enriched for SEZ6 positive tumors (64). The study provided early clinical validation of SEZ6 targeting, achieving an objective response rate (ORR) of 25%; however, development was limited by hepatotoxicity in 35% of patients, including grade ≥3 events in 12% (65).

ABBV-706, a next-generation ADC with a topoisomerase I inhibitor payload. The agent has been evaluated both as monotherapy and in combination with PD-1 inhibition in extensive-stage SCLC (NCT05599984) (66). Preliminary results from dose-escalation cohorts demonstrated encouraging activity, with an overall ORR of 43.8% (66). Grade ≥3 treatment related adverse events were primarily hematologic, including neutropenia, anemia, and leukopenia consistent with the mechanism of the topoisomerase I payload (66).

### 3.2 Non-NE lineage markers

#### 3.2.1 TROP2

TROP2 is a tumor associated surface protein expressed across multiple epithelial malignancies, including SCLC, and its expression in SCLC is enriched in non-NE tumor states, making it a relevant target in the context of lineage plasticity (67). Sacituzumab govitecan (SG), a TROP2-directed ADC carrying the active metabolite SN-38, has demonstrated clinical efficacy across several solid tumors. A phase II trial evaluated SG in 43 patients with extensive-stage SCLC progressing after platinum-based chemotherapy and immunotherapy. The study showed an ORR of 41.9%, with activity observed in both platinum-resistant (ORR 35%) and platinum-sensitive (ORR 47.8%) subgroups (64).

Sacituzumab tirumotecan (sac-TMT) is a next-generation TROP2-directed ADC that uses the same humanized anti-TROP2 antibody as SG but incorporates a more stable sulfonyl-pyrimidine linker and a belotecan-derived topoisomerase I inhibitor payload (KL610023), allowing improved plasma stability and more controlled payload release (68). It is currently being evaluated in a phase I/II first-in-human trial in advanced solid tumors, including an expansion cohort for SCLC (NCT04152499) (69).

### 3.3 Lineage-agnostic

#### 3.3.1 B7-H3

B7-H3 (CD276) is a B7-family immunoregulatory surface molecule aberrantly expressed on tumor cells in approximately 65% of SCLC cases and is associated with immune evasion, aggressive clinical behavior, and poor survival (70). Transcriptomic analysis of over 1700 tumors demonstrated consistently high B7-H3 expression across NE and non-NE lineages, unlike lineage-restricted targets such as DLL3 and SEZ6 (71). B7-H3 expression did not correlate with T cell signatures but instead associated with immunoregulatory and myeloid features including TIM-3, PD-L2, CD86, and M2 macrophage programs (71).

Ifinatamab deruxtecan (I-DXd) is a B7-H3 targeted ADC composed of an anti-B7-H3 monoclonal antibody linked to a topoisomerase I inhibitor via a tetrapeptide cleavable linker (72). In the phase II IDeate-Lung01 dose-optimization comparing I-DXd 8 vs 12 mg/kg (q3w), 12 mg/kg was selected for further study (73). At this dose, activity was observed in previously treated ES-SCLC, with a confirmed ORR of 48.2% and a median time to response of ~1.4 months (73). Responses were observed in patients with brain metastases. Toxicities were consistent with other deruxtecan-based ADCs, with cytopenias and gastrointestinal adverse events common; however, interstitial lung disease (ILD) was clinically significant, occurring in 12.4% of patients, including grade ≥3 events in 4.4%, and treatment-related mortalities (73). The phase III IDeate-Lung02 trial is currently comparing I-DXd with physician’s choice chemotherapy in relapsed SCLC (NCT06203210). In addition, the phase Ib/II IDeate-Lung03 study is evaluating I-DXd in combination with atezolizumab maintenance following standard induction therapy, as well as I-DXd combined with atezolizumab and carboplatin in the frontline setting (74).

Vobramitamab duocarmazine (MGC018) is another B7-H3 directed ADC which employs a cleavable valine–citrulline linker to deliver a duocarmycin-class DNA minor-groove alkylator (75), being explored in a relapsed ES-SCLC (NCT06227546).

#### 3.3.2 GD2

Disialoganglioside, GD2 is expressed in about 39% of SCLC, exhibiting marked intra- and inter-tumoral heterogeneity, distinguishing it from more lineage antigens (76). This variability is consistent with context dependent regulation of ganglioside biosynthetic programs and epigenetic modulation of GD2 expression (77). A randomized study of the anti-GD2 antibody dinutuximab combined with irinotecan failed to improve outcomes in relapsed SCLC (77). Although GD2-specific CAR-T approaches show cytotoxic activity in preclinical systems and epigenetic strategies can enhance GD2 expression in antigen low tumors, current evidence supports GD2 as a biologically plausible but clinically constrained target, likely requiring stringent biomarker selection and combinatorial strategies to overcome antigen heterogeneity (78–80).

## 4 Immune modulation

Incorporation of ICI into first-line carboplatin-etoposide chemotherapy has modestly but reproducibly improved survival outcomes in ES-SCLC. In the phase III IMpower133 trial, the addition of atezolizumab to CE significantly prolonged OS to 12.3 months from 10.3 months (77). Similarly, the CASPIAN trial adding durvalumab to CE improved median OS to about 13.0 vs 10.3 months (HR 0.73) (81). These studies established chemoimmunotherapy as the frontline standard; however, durable immune control is achieved in only a minority of patients.

This biology also contextualizes the repeated failure of additional checkpoint layering in unselected ES-SCLC. In the phase III SKYSCRAPER-02 trial, adding the anti-TIGIT antibody tiragolumab to atezolizumab plus chemotherapy did not improve OS or PFS (82). Similarly, the phase III KEYVIBE-008 study evaluating pembrolizumab with the anti-TIGIT antibody vibostolimab plus chemotherapy failed to meet its OS endpoint and was terminated early for futility (83). LAG-3, another inhibitory receptor co-expressed with PD-1 on exhausted T cells, is detectable in a subset of SCLC tumors (84, 85). However, no clinical trials have yet demonstrated clear benefit from LAG-3 blockade in SCLC. Together, these results suggest that in most SCLC, dominant barriers such as limited T cell infiltration and defective priming, outweigh incremental relief of inhibitory receptor signaling (86, 87).

## 5 Epigenetic priming to stabilize lineage and enhance immunogenicity

Epigenetic dysregulation underlies the remarkable lineage plasticity of SCLC and contributes to treatment resistance and immune evasion. Targeting chromatin regulators such as LSD1, EZH2, and BET proteins therefore offers an opportunity to reprogram tumor identity, restore antigen presentation, and sensitize tumors to immunotherapy.

### 5.1 BET

BET family proteins, particularly BRD4, function as chromatin “readers” that sustain oncogenic transcriptional programs and reinforce NE lineage states (88). BET inhibition preferentially disrupts BRD4-dependent NEUROD1 transcriptional activation, suppressing core neuroendocrine circuitry and impairing tumor growth in preclinical models (89). Clinically, however, pan-BET inhibitors as monotherapy have been limited by a narrow therapeutic index, most notably thrombocytopenia (90). These limitations have shifted development toward MYC-enriched patient selection and rational combinations. Preclinical models demonstrate NK cell dependent synergy between BET and HDAC6 inhibition, supporting combination approaches that may improve therapeutic index and efficacy (91, 92).

### 5.2 LSD1 (KDM1A)

LSD1 is a central epigenetic regulator of lineage state and immune evasion in SCLC. Preclinical inhibition of LSD1 with bomedemstat enhances tumor immunogenicity, increases intratumoral CD8^+^ T cell infiltration, and sensitizes tumors to PD-1 blockade, supporting its role as an immune priming strategy (93). Clinically, LSD inhibitor CC-90011 combined with nivolumab showed limited overall activity (ORR ~10%) in heavily pretreated SCLC; however, durable complete and partial responses in a subset of patients provide proof of concept for epigenetic sensitization to ICI (94).

### 5.3 EZH2

EZH2, the catalytic subunit of the Polycomb Repressive Complex 2 (PRC2), mediates trimethylation of histone H3 at lysine 27 (H3K27me3), a repressive chromatin mark that enforces transcriptional silencing (95). EZH2 is highly expressed in SCLC and contributes to immune evasion. Mechanistic studies describe a CRACD-EZH2-MHC-I axis in which EZH2 represses antigen-presentation genes, leading to CD8^+^ T cell exclusion; pharmacologic inhibition restores MHC-I expression and suppresses tumor growth in relevant models (96) (97).

In a phase I/II study of EZH1/2 inhibitor valemetostat combined with irinotecan in recurrent SCLC, objective responses occurred in 21% of patients, although durability was limited (median duration of response 4.6 months) (26). Importantly, on-treatment biopsies demonstrated increased MHC-I expression and shifts in subtype markers, consistent with epigenetic remodeling (26). These findings support EZH2 inhibition as a biologic priming strategy and provide rationale for combination with immunotherapy (26). EZH2 also contributes to chemotherapy resistance by repressing DNA damage response genes such as *SLFN11* (98, 99).

## 6 Poly ADP ribose polymerase inhibition

SCLC is characterized by marked genomic instability, high tumor mutational burden, and profound dependence on DDR pathways (100). PARP plays a central role in the repair of single strand DNA breaks, and inhibition of PARP leads to the accumulation of unrepaired DNA damage, replication stress, resulting in tumor cell death (101). Clinical trials evaluating PARP inhibitors in SCLC have yielded largely negative results. In a phase II study, adding veliparib to temozolomide significantly improved ORR (39% vs 14%) but did not significantly prolong PFS (4 month PFS 36% vs 27%; median PFS 3.8 vs 2.0 months) (102). Similarly, olaparib plus temozolomide achieved a response rate of 41.7% with median PFS of 4.2 months and OS of 8.5 months, indicating activity in selected patients (103). Frontline randomized trials combining PARP inhibition with platinum-etoposide chemotherapy have also shown minimal benefit. In a phase II study, veliparib added to chemotherapy yielded comparable outcomes to chemotherapy alone (median PFS 5.8 vs 5.6 months; OS 10.1 vs 12.4 months) without meaningful improvement in response, while increasing hematologic toxicity (5, 104).

PARP inhibitors were also combined with immune checkpoint blockade, based on the premise that DDR inhibition may increase cytosolic DNA signaling and immune visibility. In a single arm phase II study of durvalumab plus olaparib in relapsed SCLC, the combination was feasible but showed limited activity, with an ORR of 10.5% (105). In a randomized phase II maintenance study of atezolizumab with or without talazoparib following induction chemoimmunotherapy, patients with SLFN11 positive tumors derived greater benefit: median PFS was approximately 4.2 months with the combination versus 2.8 months with atezolizumab alone in the SLFN11 positive subgroup, whereas no meaningful benefit was observed in SLFN11 negative tumors. This modest PFS improvement came at the cost of substantially higher grade ≥3 hematologic toxicity (50% vs 4%), driven largely by grade 3 anemia (37% vs 2%) and additional thrombocytopenia (25% vs 0%) (106).

## 7 Monitoring

### 7.1 Biomarker guided treatment adaptation and longitudinal monitoring

As in hematologic malignancies, longitudinal blood-based monitoring may provide an early readout of residual disease and impending relapse (107). SCLC ctDNA levels provide strong prognostic information (108). A meta-analysis of 391 patients across nine cohorts demonstrated that higher ctDNA burden correlated with worse progression free and overall survival (109). Longitudinal monitoring shows detectable ctDNA after curative-intent therapy predicts relapse, while dynamic changes correlate with chemotherapy response (110). Recent longitudinal ctDNA studies have extended these observations in SCLC.

In extensive-stage disease, early ctDNA dynamics correlate with treatment response and outcomes (111, 112). More recently, limited-stage studies suggest that serial ctDNA monitoring during and after chemoradiotherapy may help refine relapse risk and identify patients most likely to benefit from consolidation immunotherapy (113). In one study, baseline copy number amplifications disappeared after response but re-emerged at progression in most patients. In one study, baseline copy number amplifications disappeared after response but re-emerged at progression in most patients (114). Because actionable driver mutations are uncommon in SCLC and resistance often reflects non genetic state transitions, molecular features derived from cfDNA and cfDNA epigenomics offer a more practical strategy for longitudinal disease tracking (115, 116).

Methylation profiling has emerged as an informative modality. Lower cfDNA methylation scores correlate with earlier stage and improved outcomes (117). Genome wide methylation analysis distinguished SCLC patients from non-cancer individuals with high accuracy (AUC 0.99 for limited-stage and 1.0 for extensive-stage disease), outperforming copy-number (117). Large datasets further show that SCLC methylation patterns resemble other neuroendocrine tumors rather than NSCLC, independent of anatomical site (118). Using cfMeDIP-seq, two methylome-defined groups with significantly different survival outcomes (median OS 21 vs 12 months) were identified, indicating that cfDNA methylation profiling can serve as a practical prognostic biomarker in SCLC (118).

Emerging cfDNA approaches further capture tumor biology. Nucleosome footprinting detecting transcription factor binding (REST, NEUROD1, ASCL1) correlates with immunotherapy response, and lineage state (119). Fragmentation profiling using the DELFI model distinguished SCLC from healthy individuals with 96% accuracy, demonstrating the diagnostic potential of cfDNA fragmentomics (120, 121). More recently, plasma H3K4me3 cfChIP seq has demonstrated that circulating nucleosomes retain tumor specific epigenetic information, enabling noninvasive assessment of tumor burden and transcriptional lineage programs (121). In SCLC, plasma derived chromatin signatures accurately recapitulated key subtype defining transcription factors including ASCL1, NEUROD1, and POU2F3 and correlated strongly with matched tumor RNA expression, supporting the feasibility of blood-based molecular subtyping and longitudinal monitoring of tumor state (121).

Despite these advances, cfDNA approaches face challenges, including cross-platform variability, uncertain thresholds, and difficulty separating tumor derived from normal cfDNA signals, as ikon well as uncertainty regarding which molecular features should guide treatment decisions (114). Progress toward clinical utility will likely require prospective trials incorporating cfDNA monitoring and the development of shared longitudinal datasets analogous to TCGA to enable iterative biomarker refinement.

### 7.2 Extrachromosomal DNA

Interest has also focused on extrachromosomal DNA (ecDNA), circular chromatin structures carrying amplified oncogenes that enable rapid evolutionary adaptation (122, 123). ecDNA is present in ~19% of SCLC tumors and frequently harbors MYC-family amplifications (124). In patient-derived xenograft models, ecDNA was detected in 45% of cases and was enriched in relapsed disease and chemotherapy resistance (124, 125). By enabling high copy, dynamically regulated oncogene amplification outside chromosomal constraints, ecDNA may represent both a biomarker of aggressive biology and a mechanistic driver of therapeutic resistance. Importantly, Pongor et al. showed that ecDNA associated focal amplifications can be detected non-invasively in plasma using cfChIP seq of H3K4me3 marked cell free nucleosomes (124).

## 8 Emerging issues

### 8.1 ADC induced pneumonitis

Target density is frequently heterogeneous within and across SCLC tumors, limiting the efficacy of ADCs that depend on efficient target binding and internalization (126). Spatial variability in antigen expression, compounded by lineage plasticity and NE state transitions, increases the likelihood that antigen-low or antigen-negative subclones escape direct targeting (127). Top-I inhibitor based ADCs partially address this constraint through bystander effect, whereby payload released from target-high internalizing cells can diffuse into adjacent tumor cells independent of antigen density, extending cytotoxicity across heterogeneous tumors (128).

This design, however, introduces a safety trade off. The same membrane permeability that enables bystander activity can increase off-tumor exposure, making pulmonary toxicity, particularly pneumonitis/ILD, a dose- and exposure-dependent risk (129, 130). Risk is further influenced by baseline lung vulnerability. Prior thoracic radiotherapy can induce persistent subclinical alveolar epithelial injury, lowering the threshold for subsequent drug related lung damage (131, 132). Cumulative payload exposure, linker dependent release kinetics, and radiation primed lung tissue may therefore converge to increase susceptibility to ILD.

Clinical experience supports this mechanistic concern. Early phase I evaluation of the DLL3-directed Top-I ADC ZL-1310 has reported cases of ILD; however, these findings are currently based on company press releases, and peer-reviewed data are not yet available (133). Deruxtecan containing ADCs have established ILD as a clinically meaningful toxicity (12.4% of patients, including fatal events), reflected in FDA label warnings (134). By contrast, SN-38 based ADCs have not shown a comparable signal in large datasets, indicating that pulmonary risk is not solely determined by Top-I class but is influenced by payload chemistry, linker stability, biodistribution, and release kinetics.

As bystander enabled ADCs are increasingly used to overcome antigen heterogeneity, prospective capture of thoracic radiotherapy history, conservative dose escalation in higher-risk populations, and structured ILD surveillance will be essential to mitigate compounded lung injury risk (130).

### 8.2 T cell fitness and microenvironmental barriers as limiters of TCE

Experience from hematologic malignancies, where T cell engagers have been in use for over a decade, provides a useful framework for understanding resistance mechanisms that may emerge with such therapies in SCLC (135).

Clinical experience with CD19 TCE therapy in B-ALL has established a clear distinction between primary resistance and acquired resistance (135, 136). Primary resistance most often is associated with an unfavorable baseline disease and immune context with impaired effector target balance (137). In contrast, acquired resistance following an initial response frequently reflects reduced/absent surface CD19 (“CD19 negative relapse”). More rarely, lineage switch to AML has been demonstrated as an escape mechanism following CD19-directed therapy (138, 139). A similar framework applies in multiple myeloma treated with BCMA TCEs. Primary resistance is associated with an unfavorable baseline immune state, including a higher proportion of phenotypically exhausted T cells and reduced functional reserve (140, 141). In relapsed/refractory myeloma baseline risk scores incorporating marrow reserve and systemic inflammation can predict inferior responses (142). Acquired resistance, by contrast has been linked to deterioration of T cell fitness, in which sustained engager mediated activation drives exhaustion, even when antigen expression is retained (143).

**Figure 3:**
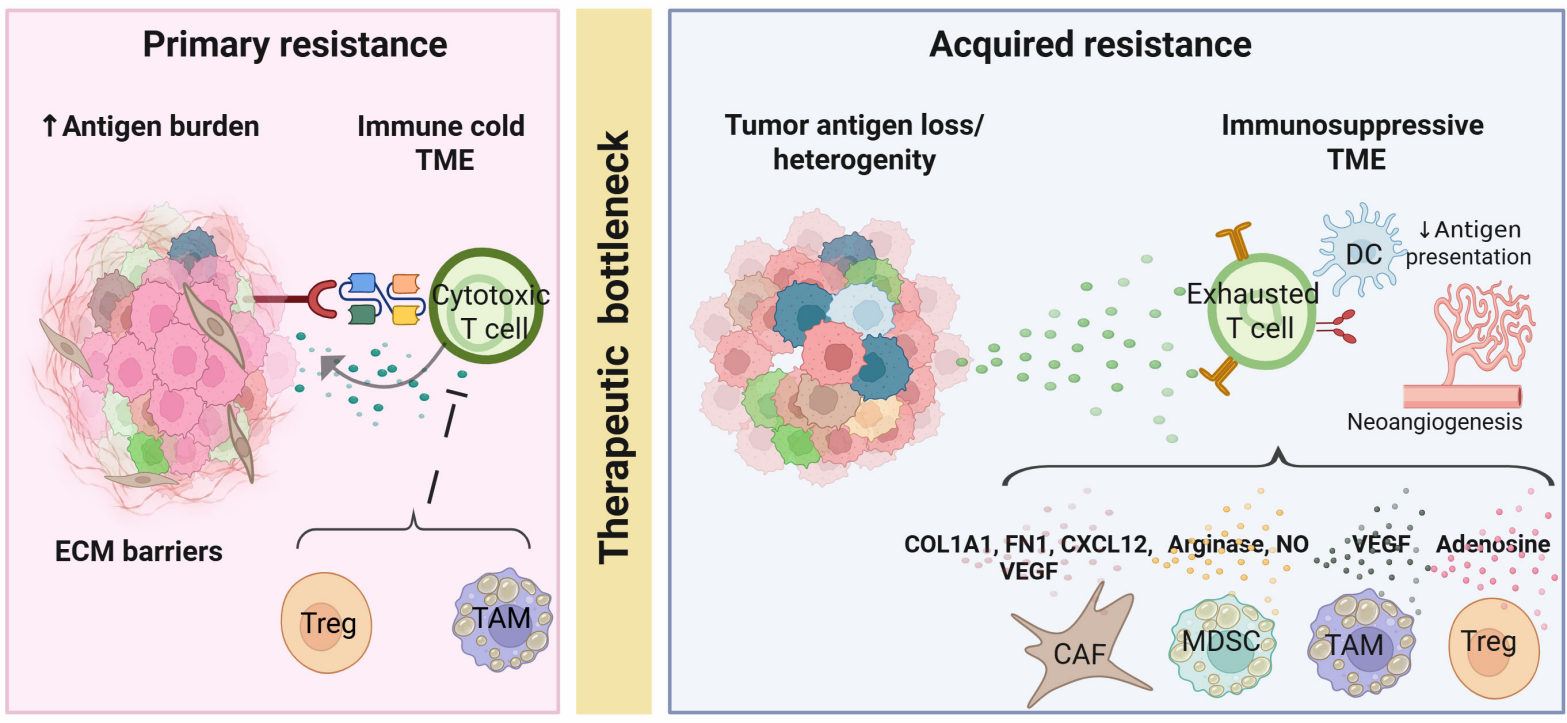
Mechanisms of primary and acquired resistance to T cell engagers in SCLC. Left: Primary resistance is characterized by an immune cold tumor microenvironment (TME) and elevated antigen burden. Physical barriers imposed by extracellular matrix (ECM) and suppressive immune subsets including regulatory T cells (Tregs) and M2-polarized macrophages, limit effective cytotoxic T cell infiltration and function. Right: Acquired resistance emerges through tumor heterogeneity and adaptive remodeling of the TME. Clonal selection and tumor cell plasticity contribute to immune evasion, while metabolic byproducts and reduced antigen presentation promote T-cell exhaustion. Stromal and myeloid populations including cancer associated fibroblasts (CAFs), myeloid derived suppressor cells (MDSCs), tumor-associated macrophages (TAMs), and Tregs, reinforce an immunosuppressive niche through production of extracellular matrix components (COL1A1, FN1), chemokines (CXCL12), angiogenic factors (VEGF), and metabolic suppressors (arginase, nitric oxide, adenosine). Together, these mechanisms establish a therapeutic bottleneck that constrains durable antitumor immunity.

In solid tumor models, preclinical studies highlight additional resistance barriers that are less prominent in hematologic malignancies (**Figure 3**). Low baseline intratumoral T cell density has been shown to associate with primary resistance to TCEs (144). Beyond immune exclusion, low and heterogeneous target antigen density represents a distinct tumor-intrinsic constraint. Low antigen density can generate insufficient TCR signaling strength and duration to support full cytokine production and proliferative programming (145). Consistent with this model, recent *in vivo* studies demonstrate that low antigen density limits TCE efficacy, and that augmenting local IL-2 signaling restores antitumor activity (146).

Suppressive myeloid and vascular programs can also impose microenvironmental barriers to TCE activity (147). Tumor associated macrophages and myeloid derived suppressor cells can attenuate TCE driven T cell responses through metabolic competition, inhibitory ligand expression, and cytokine-mediated suppression (147, 148). Concurrently, abnormal tumor vasculature restricts T cell trafficking and fosters hypoxic, nutrient-deprived niches (54). Preclinical studies indicate that myeloid reprogramming and vascular normalization strategies can enhance T cell infiltration and sensitize tumors to T cell engaging therapies (54).

Finally, adaptive immune checkpoints constrain TCE activity. TCE-driven T cell activation induces a compensatory PD-1/PD-L1 inhibitory axis, and combining a tumor-targeting CD3 bispecific with PD-L1 blockade enhances intratumoral effector CD4^+^ and CD8^+^ phenotypes and improves tumor control relative to either agent alone (149).

Despite these emerging principles, SCLC specific mechanisms of resistance to TCEs remain poorly defined and require dedicated investigation. Given the rapid clinical kinetics and profound microenvironmental remodeling characteristic of SCLC, systematic collection of pre- and posttreatment tumor samples, and where tissue is limited, longitudinal CTC based profiling (150), will be essential. Embedding deep immune and tumor profiling into early-phase SCLC TCE trials will be crucial to distinguish primary from acquired resistance, define antigen-dependent versus antigen independent escape, and identify rational combination strategies.

### 5.3 CNS metastases as a special biological compartment

CNS metastases represent a biologically distinct and therapeutically constrained compartment in SCLC. It is a major driver of morbidity, with approximately 10-24% of patients presenting with brain metastases at diagnosis and >50% developing CNS disease over the course of illness (151). Treatment response within the CNS is shaped by the blood-tumor barrier (BTB), local immune accessibility, and local CNS directed treatments.

A major barrier to therapeutic progress in SCLC CNS disease has been the lack of tractable models that faithfully recapitulate brain metastasis biology (152). However, recent studies have described models that reproducibly generate brain metastases and permit biologic and therapeutic interrogation (153). These studies also demonstrate that SCLC brain metastases undergo organ-adaptive reprogramming rather than representing simple dissemination of systemic disease. Brain-tropic derivatives exhibit transcriptional programs enriched for neuronal identity, cell–cell junctions, cytoskeletal remodeling, and extracellular matrix interactions (153).

ADCs may retain intracranial activity because established metastases develop a disrupted, variably permeable BTB that permits antibody penetration allowing cytotoxic payloads to exert local effects (154). Consistent with this framework, intracranial activity has been reported with I-DXd in patients with baseline brain metastases. T-cell engagers, in contrast, depend on immune trafficking; emerging real-world data suggest that tarlatamab can induce rapid intracranial responses, including in untreated brain metastases (155).

Radiotherapy remains central to CNS disease control (156). In limited-stage SCLC, prophylactic cranial irradiation (PCI) reduces the incidence of brain metastases and improves survival after definitive chemoradiation (157). Several phase III trials are re-evaluating PCI in the MRI era (SWOG S1827/MAVERICK, PRIMALung) comparing MRI surveillance alone or with PCI to determine whether surveillance can preserve outcomes while reducing PCI-related neurotoxicity (NCT04155034, NCT04790253). In parallel, randomized trials have focused on mitigating PCI neurotoxicity: in NRG-CC003, hippocampal avoidance (HA) during PCI maintained noninferior intracranial control while reducing overall neurocognitive toxicity (158). In extensive stage disease, PCI lowers CNS relapse rates but has not demonstrated survival benefit in the context of routine MRI surveillance, and many centers now favor MRI monitoring in responders with negative baseline imaging (159). As systemic therapies with intracranial activity emerge, optimal integration of radiation, systemic therapy, and corticosteroid management remain an active area of investigation (159, 160).

## 9 Future directions

Unlike many solid tumors in which resistance is driven by stepwise genetic evolution, SCLC is distinguished by near universal TP53 and RB1 loss and a highly plastic NE regulatory network that enables rapid, non-genetic state transitions under therapeutic pressure. These lineage shifts reshape signaling pathways, metabolic programs, and immune visibility, allowing tumor persistence despite effective initial therapy. The following sections highlight key plasticity linked mechanisms that recurrently emerge in SCLC and represent actionable targets for next generation, state-aware therapeutic strategies.

### 9.1 Epithelial to mesenchymal transition

SCLC exhibits marked phenotypic heterogeneity, including non-NE states with EMT like features that emerge under therapeutic and microenvironmental pressure (161, 162). In genetically engineered models, non-NE cells include epithelial like and mesenchymal like subpopulations, the latter with greater metastatic competence and enrichment of TGF-β signaling (163). Functionally, TGF-β pathway blockade attenuates metastasis across allograft and autochthonous RP models, supporting TGF-β signaling as a driver of metastasis-associated state programs in SCLC (163). Beyond metastasis, EMT linked TGF-β programs also intersect with therapy tolerance. Tight junction remodeling through claudin-1 (CLDN1) overexpression activates TGF-β1 dependent EMT signaling in cell lines and confers reduced sensitivity to chemotherapy (39). While pharmacologic inhibition of TGF-β/EMT pathways suppresses EMT phenotypes in lung cancer models, SCLC specific clinical validation remains limited (39).

### 9.2 Adaptive rewiring

Under therapeutic pressure, SCLC engages adaptive signaling pathways that support tumor persistence through lineage state transitions. One emerging node is focal adhesion kinase (FAK/PTK2), which integrates adhesion and mechanical cues to regulate cytoskeletal organization. A subset of SCLC models exhibits elevated phosphorylated FAK and increased sensitivity to the FAK inhibition (89). AXL receptor tyrosine kinase, activated by its ligand GAS6 (growth arrest–specific 6), is another stress and EMT-associated signaling node implicated in therapy resistance and drug-tolerant states. AXL is linked to stem-like phenotypes, consistent with non-NE transitions and drug tolerance (164). Replication stress (RS) has emerged as a potential biomarker for combined AXL and ATR inhibition in SCLC. RS-high models display heightened sensitivity to AXL inhibitor bemcentinib in combination with ATR blockade, indicating that therapy-adapted cells become co-dependent on stress signaling and checkpoint activation (165). Although not typically a dominant driver in treatment-naïve SCLC, HGF-MET activation emerges in chemotherapy resistant and relapsed disease (166, 167). Altogether, RTK pathways are better viewed as context dependent adaptive programs in SCLC rather than fixed oncogenic dependencies, arguing against unselected single-agent RTK inhibition and favoring biomarker-guided combinations designed to suppress adaptive signaling and state transitions (167, 168).

### 9.3 Metabolic rewiring and lactate-associated immune suppression

In hematologic malignancies, particularly AML metabolic exchange between malignant cells and the bone marrow niche supports tumor fitness. For example, lactate transport via MCT1/MCT4 contributes to metabolic adaptation and survival (169). Lactate also functions as an immunoregulatory signal, promoting an immune-suppressive bone marrow niche through effects on myeloid polarization and regulatory T cell accumulation (165, 170).

A conceptually similar program operates in SCLC wherein lactate exchange occurs between tumor cell states: electrically active non-NE cells export lactate that is taken up by neighboring NE cells, a state-dependent metabolic shuttle that sustains oxidative metabolism and inter-state cooperation (171). Clinically, this wiring is reflected in the longstanding prognostic value of serum LDH, consistently associated with poorer survival in both limited and extensive-stage disease (172). Preclinical studies further show heterogeneous expression of monocarboxylate transporters, and MCT1 inhibition is effective in MCT1-high/MCT4-low tumors (173).

Metabolic rewiring is further reinforced by oncogenic context. MYC driven SCLC states display enhanced glycolytic programming (42). Moreover, glycolytic reprogramming can promote immune evasion through an LDHA-lactylation-PRC2-MHC-I axis, in which lactate driven histone lactylation suppresses antigen presentation and reduces CD8^+^ T cell recognition (174). Collectively, metabolic plasticity is an adaptive program integrating lineage cooperation, tumor persistence, and immune suppression, supporting new therapeutic strategies that jointly target metabolic flux and antitumor immunity (174).

## 10 Conclusions

Future therapeutic progress in SCLC will require biomarker-guided strategies to account for lineage identity, antigen heterogeneity, metabolic state and immune context. In hematologic malignancies, durable advances have emerged by targeting convergent survival and stress-response dependencies including BCL-2 family programs, replication stress vulnerabilities, and epigenetic state-control circuitry. A similar mechanism-based organization of therapy might benefit SCLC, where tumor fitness is sustained not by a single dominant oncogene, but by adaptive lineage plasticity and stress-response networks. Given the near universal loss of TP53 in SCLC and the limited feasibility of direct p53 restoration, therapeutic efforts are more realistically directed toward exploiting stress response, replication, and metabolic dependencies that arise from p53 deficiency (13).

Promising directions include temporally sequenced combination regimens that anticipate plasticity driven escape and adapt therapy to evolving lineage, antigen, and immune landscapes. These would integrate (i) state-modulating priming (e.g., epigenetic interventions that enhance antigen presentation and immune visibility), (ii) dual or sequential antigen targeting that pairs lineage linked antigens with lineage-decoupled targets, (iii) cytotoxic or DDR/replication-stress perturbation, and (iv) metabolic reprogramming approaches that disrupt NE/non-NE metabolic cooperation while improving effector T cell fitness and countering immune suppression within the tumor microenvironment.

## Glossary

ADC: Antibody–drug conjugate
AE: Adverse event
ALL: Acute lymphoblastic leukemia
AML: Acute myeloid leukemia
ASCL1: Achaete-scute homolog 1
ATR: Ataxia telangiectasia and Rad3-related
AUC: Area under the curve
AXL: AXL receptor tyrosine kinase
B-ALL: B-cell acute lymphoblastic leukemia
BCMA: B-cell maturation antigen
BET: Bromodomain and extraterminal domain
BCL-2: B-cell lymphoma 2
BCL-XL: B-cell lymphoma-extra-large
BRD4: Bromodomain-containing protein 4
BTB: Blood–tumor barrier
CAF: Cancer-associated fibroblast
CAR-T: Chimeric antigen receptor T cell
CE: Carboplatin–etoposide
cfChIP-seq: Cell-free chromatin immunoprecipitation sequencing
cfDNA: Cell-free DNA
cfMeDIP-seq: Cell-free methylated DNA immunoprecipitation sequencing
CLDN1: Claudin-1
CNS: Central nervous system
CRACD: Capping protein inhibiting regulator of actin dynamics
CRS: Cytokine release syndrome
CTC: Circulating tumor cell
ctDNA: Circulating tumor DNA
DDR: DNA damage response
DELFI: DNA evaluation of fragments for early interception
DLL3: Delta-like ligand 3
DNMT1: DNA methyltransferase 1
ecDNA: Extrachromosomal DNA
EMT: Epithelial-to-mesenchymal transition
ES-SCLC: Extensive-stage small cell lung cancer
EZH2: Enhancer of zeste homolog 2
FAK: Focal adhesion kinase
GAS6: Growth arrest–specific 6
GD2: Disialoganglioside 2
HDAC: Histone deacetylase
HGF: Hepatocyte growth factor
H3K27me3: Histone H3 lysine 27 trimethylation
H3K4me3: Histone H3 lysine 4 trimethylation
ICI: Immune checkpoint inhibitor
ICANS: Immune effector cell-associated neurotoxicity syndrome
ILD: Interstitial lung disease
JAK: Janus kinase
LAG-3: Lymphocyte-activation gene 3
LDH: Lactate dehydrogenase
LDHA: Lactate dehydrogenase A
LSD1: Lysine-specific demethylase 1 (KDM1A)
MCL-1: Myeloid cell leukemia 1
MCT1/4: Monocarboxylate transporter 1/4
MDS: Myelodysplastic syndrome
MET: Mesenchymal–epithelial transition factor
MHC-I: Major histocompatibility complex class I
MRD: Minimal residual disease
MYC: MYC proto-oncogene
NE: Neuroendocrine
non-NE: Non-neuroendocrine
NSCLC: Non-small cell lung cancer
ORR: Objective response rate
OS: Overall survival
PARP: Poly (ADP-ribose) polymerase
PCI: Prophylactic cranial irradiation
PD-1: Programmed cell death protein 1
PD-L1: Programmed death-ligand 1
PFS: Progression-free survival
PRC2: Polycomb repressive complex 2
RS: Replication stress
RTK: Receptor tyrosine kinase
SCLC: Small cell lung cancer
SCLC-SL: Stem-like small cell lung cancer subtype
SEZ6: Seizure-related homolog protein 6
SLFN11: Schlafen family member 11
SN-38: 7-ethyl-10-hydroxycamptothecin
SWI/SNF: SWItch/sucrose non-fermentable chromatin remodeling complex
TAM: Tumor-associated macrophage
TCE: T-cell engager
TGF-β: Transforming growth factor beta
TIGIT: T cell immunoreceptor with Ig and ITIM domains
TIM-3: T cell immunoglobulin and mucin-domain containing 3.
